# The neighbourhood social environment and alcohol use among urban and rural Scottish adolescents

**DOI:** 10.1007/s00038-018-1181-8

**Published:** 2018-12-03

**Authors:** Gina Martin, Joanna Inchley, Alan Marshall, Niamh Shortt, Candace Currie

**Affiliations:** 1 Child and Adolescent Health Research Unit, School of Medicine, University of St Andrews, St Andrews, KY16 9TF UK; 2 Social Policy, School of Social and Political Science, University of Edinburgh, Edinburgh, EH8 9LD UK; 3 Centre for Research on Environment, Society and Health, School of GeoSciences, University of Edinburgh, Edinburgh, EH8 9XP UK

**Keywords:** Adolescents, Alcohol, Neighbourhood, Cross-classified, Multilevel, Urban, Rural, Alcohol outlet density, Social cohesion, Disorder

## Abstract

**Objectives:**

This research examined the relationship between neighbourhood social environmental characteristics and drinking outcomes among a sample of urban and rural adolescents.

**Methods:**

From a sample of 1558 Scottish secondary schoolchildren, surveyed as part of the 2010 Health Behaviour in School-aged Children study, we modelled three drinking outcomes on a variety of neighbourhood conditions, including social cohesion, disorder, alcohol outlet density, deprivation, and urban/rurality. Nested and cross-classified multilevel logistic regressions were specified.

**Results:**

An urban-to-rural gradient was found with non-urban adolescents exhibiting higher odds of having ever drank. Neighbourhood social cohesion related to having ever drank. Among drinkers, those living in accessible small towns had higher odds of weekly drinking and drunkenness compared to urban areas. Higher odds of drunkenness were also found in remote rural areas. Those residing in the least deprived areas had lower odds of weekly drinking.

**Conclusions:**

In Scotland, inequalities exist in adolescent alcohol use by urban/rurality and neighbourhood social conditions. Findings support regional targeting of public health efforts to address inequalities. Future work is needed to develop and evaluate intervention and prevention approaches for neighbourhoods at risk.

**Electronic supplementary material:**

The online version of this article (10.1007/s00038-018-1181-8) contains supplementary material, which is available to authorized users.

## Introduction

Adolescent alcohol use is an identified public health concern. Scotland’s 15-year-olds rank fifth of 41 countries in Europe for having been drunk at least twice (Inchley et al. 2016). Further, it has been estimated that 15 individuals per day aged 17 or under are admitted to Scottish hospitals intoxicated (Christie 2008; NHS Quality Improvement Scotland 2008); this equates to approximately 1707 hospital admissions per 100,000 of the population aged 13–17, annually. These patterns are of concern given the wide array of harms associated with alcohol use among adolescents.

Because initiation into alcohol use often occurs in adolescence, this life stage has been established as a crucial period to reduce drinking (Kuntsche et al. 2005). Consequently, it is important to understand the risk and protective factors associated with alcohol use in order to develop targeted public health policies (Bryden et al. 2013). Evidence suggests adolescent alcohol use varies across neighbourhoods (Fagan et al. 2015; Jackson et al. 2016). However, which specific neighbourhood characteristics underlie this variation is not fully understood (Fagan et al. 2015).

Many features of the neighbourhood have been theorised to be associated with adolescent alcohol use (Fagan et al. 2015). Studies examining neighbourhood socio-economic factors have found mixed results (Bryden et al. 2013; Jackson et al. 2014), thus implying that more research is required to examine neighbourhood social factors. Neighbourhood social conditions, such as cohesion and collective efficacy, have drawn more recent attention and are often posited to underlie the relationship between neighbourhood economic conditions and alcohol use (Fagan et al. 2013; Jackson et al. 2014). Theories of the social environment and substance use suggest that the positive bonds in society deter adolescents from substance use (Wray-Lake et al. 2012),while neighbourhoods with greater disorder may encourage alcohol use as a way of coping with environmental stress (Hill and Angel 2005). However, reviews of neighbourhood social factors and drinking behaviour among adolescents indicate varied findings (Bryden et al. 2013; Jackson et al. 2014). This may, in part, reflect equivocal measurements of the social environment (Martin et al. 2017a) and/or different drinking outcomes included in these studies.

Research examining neighbourhood characteristics and adolescent drinking typically focuses on urban environments (Bryden et al. 2013). However, adolescents’ urban/rural status has been found to associate with their alcohol use and has been theorised to contribute to geographic variation in drinking behaviours (Slutske et al. 2016). Contemporary research has shown that adolescents residing in rural areas tend to drink alcohol at higher rates than those in urban areas (Dixon and Chartier 2016; Donath et al. 2011). The mechanisms behind this are not well understood but may be due to physical and/or cultural differences that exist between these communities (Donath et al. 2012).

There has also been much interest in the associations between commercial alcohol availability and adolescent alcohol use. Bryden et al. (2012) report that the evidence is inconclusive regarding these relationships. Increased availability may make alcohol purchasing easier through greater physical access and reduced prices, due to market competition (Shortt et al. 2018; Treno et al. 2013). However, as it is often illegal to sell alcohol to someone under a certain age, 18 in Scotland, the presence of outlets does not necessarily mean alcohol is easily available. More likely, a higher density of alcohol outlets may influence adolescent alcohol use via neighbourhood social norms and the normalisation of alcohol consumption (Kuntsche et al. 2008; Shortt et al. 2018). Maimon and Browning (2012) found that those residing in areas with higher alcohol outlet density and lower collective efficacy had higher predicted probability of alcohol use. It is important to consider alcohol availability as an important covariate in order to avoid biased conclusions about the influence of the social characteristics of the neighbourhood on alcohol use (Mohnen et al. 2011). This is particularly relevant given that more alcohol outlets tend to be present in areas of both higher deprivation and lower social capital (Shortt et al. 2015; Theall et al. 2009).

Results from neighbourhood studies that only assess neighbourhood variation may be misleading if variation from other contexts, such as school, are ignored (De Clercq et al. 2014; Dunn et al. 2015). Studies that examined adolescent smoking, using cross-classified multilevel models to account for the influence of non-nested contexts (where individuals are nested in schools and neighbourhoods, but schools are not necessarily nested within neighbourhoods or vice versa), found that neighbourhood effects are overestimated when ignoring school-level variation (De Clercq et al. 2014; Dunn et al. 2015).

This paper aims to address the following questions:

1. To what extent does adolescent alcohol use vary by neighbourhood?
2. Are there associations between neighbourhood characteristics and adolescent alcohol use?

## Method

### Participants

Survey data were collected as part of the 2010 Scottish Health Behaviour in School-aged Children (HBSC) survey, a World Health Organisation cross-national study (Currie et al. 2009). The 2010 Scottish survey of pupils in the fourth year of secondary school (S4: approximately 15 years old) included a boosted sample of rural schools allowing for comparisons at various levels of urban/rurality (Levin et al. 2014). Ethical approval was granted by the University of Edinburgh’s School of Education Ethics Committee. Prior informed consent was also obtained at local authority, school, parent, and pupil levels.

Pupils reported their residential postcode. In Scotland, postcodes represent a small geographical area making it possible to geocode (assign latitudes and longitudes) to each adolescent. Scottish data zones (DZ) and intermediate data zones (IDZs) are higher levels of geography which contain multiple postcodes. DZs (of which there are 6505) have on average 750 residents. IDZs are built up from data zones, representing 1235 regions in Scotland, containing on average 4000 residents. IDZs were developed based on administrative data and local knowledge (Flowerdew and Feng 2004). When linking in alcohol outlet densities (AOD), urban/rurality and neighbourhood deprivation, the finest geographic resolution available was used to achieve the most detailed estimate.

To increase the reliability of neighbourhood-level measures derived from aggregated individual-level responses (neighbourhood social cohesion and neighbourhood disorder), the sample was limited to 1561 students who reported their postcode and resided in an IDZ with 5 or more students (Martin et al. 2017b; Prins et al. 2014). Those included in the study were significantly (*p* < 0.05) more likely to be in the high-family-affluence tertile and to report their ethnicity as white, than those excluded, but were no more likely (*p* > 0.05) to be male, have ever drank, drink weekly, or have been drunk twice or more. An additional three students were removed from analysis based on inconsistent responses on the alcohol use questions.

### Measures

#### Drinking behaviours

Three drinking behaviours were considered in these analyses. (1) *Ever drank* was classified as those who reported an age at which they had first drunk alcohol (“more than a small amount”) as opposed to “never”. (2) *Weekly drinking* was calculated by the following question: “at present how often do you drink anything alcoholic, such as beer, wine, or spirits? Try to include even those times when you only drank a small amount” Responses included frequency of consumption (every day, every week, every month, hardly ever, and never). Those who reported drinking any beverages daily or weekly were classified as weekly drinkers. (3) *Drunkenness* was assessed with the following question: “have you ever had so much alcohol that you were really drunk?” Responses were: never, once, two–three times, four–ten times, more than ten times. This was dichotomised into less than twice or twice or more.

#### Demographics and family characteristics

Sex was included based on self-report to the question: are you a boy or a girl?

Although all students were approximately the same age (15), even small age differences may impact behaviour, given the vast number of biological and social changes that occur during this time period. Age was based on year and month of birth.

Adolescent family structure was classified as living in a family with (1) both parents, (2) a single-parent household, or (3) with a step-parent family or other family composition (Levin and Currie 2010).

Family affluence was measured on a composite scale (Currie et al. 2008) using responses to four questions regarding family vehicle and computer ownership, having one’s own bedroom, and family holidays. The items were combined using categorical principal components analysis to create tertiles of low, medium, and high family affluence, for the total sample using CATPCA in SPSS, as recommended by Batista-Foguet et al. (2004) (see Levin et al. 2014). Family affluence has been found to be associated with adolescent drinking outcomes (Obradors-Rial et al. 2018).

Respondents reported their ethnic background(s). This was dichotomised into (1) white only, or (2) other, due to there being a small number of individuals within the sample, who identified as non-white.

#### Residential characteristics

Data were obtained from the Centre for Research on Environment, Society and Health at the University of Edinburgh who geocoded all alcohol outlets that held a licence to sell alcohol in 2012 based on postcodes. These data were used to estimate a measure of AOD using Kernel Density Estimation (KDE). This process divided Scotland into 100 × 100 m grid cells and assessed the number and proximity of outlets at a radius of the centre of each cell. Outlets nearer the centre were given greater weight than those further away; therefore, the value represents a proximity-weighted estimate of the density of each outlet type. See Supplemental Material for more details on KDE. Data were classified as on-trade (e.g. bars or restaurants) and off-trade (e.g. shops) (Shortt et al. 2018). As a first step, the models were run with an 800 m radius as this approximately equates to a 10-min walk (Shortt et al. 2016). Sensitivity analysis was also conducted using the 400 and 1000 m radius.

Neighbourhood socio-economic condition was determined by the income domain of the Scottish Index of Multiple Deprivation (SIMD) 2012. This measure was based on quintiles of all DZs in Scotland. The SIMD consists of seven domains, such as access to services and crime. Using the income domain as the most appropriate indicator of neighbourhood socio-economic circumstances follows the precedent of past studies (Levin et al. 2014; Shortt et al. 2015). The two most deprived categories were combined, as few of the sample (8%) resided in the most deprived quintile.

Respondents’ home postcodes were used to classify urban/rurality. Urban/rurality was classified into six categories: (a) large cities (population of 125,000 or more), (b) other urban (population ≥ 10,000 and < 125,000), (c) accessible towns (population between 3000 and 9999 and within a 30-min drive to a settlement ≥ 10,000), (d) remote towns (population between 3000 and 9999 and more than a 30-min drive to a settlement of ≥ 10,000, (e) accessible rural (population < 3000 and within a 30-min drive to a settlement of ≥ 10,000), and (f) remote rural (population < 3000 and more than a 30-min drive to a settlement ≥ 10,000).

Neighbourhood social cohesion was measured using three questions from the HBSC survey: in the area where you live (1) you can trust people around here, (2) people say “hello” and talk to each other in the streets, and (3) it is safe for younger children to play outside. Responses ranged from “agree a lot” to “disagree a lot”, on a five-point scale. The Cronbach’s alpha at the individual level (perceived social cohesion) was 0.745. Neighbourhood disorder was measured using the same procedure. Three questions were used in this measure: in the area where you live are there (1) groups of young people who cause trouble? (2) litter, broken glass or rubbish lying around?, and (3) run-down houses or buildings? Responses ranged from “none” to “lots”, on a three-point scale. The Cronbach’s alpha at the individual level (perceived disorder) was 0.754. Both measures have previously shown reliability (items were found to highly correlate) using exploratory factor analysis (EFA) and confirmatory factor analysis (CFA) and showed measurement invariance between urban/rural classifications (Martin et al. 2017b).

Neighbourhood-level aggregation occurred using a three-level item response model accounting for item severity and the respondent’s sex (Martin et al. 2017b). The reliability at the neighbourhood level for social cohesion and disorder was 0.577 and 0.563, respectively. These measures have shown convergent validity (Martin et al. 2017b).

### Statistical analysis

Analysis was conducted to examine whether the three adolescent drinking outcomes did indeed vary by neighbourhood (IDZs) (Research Question 1). This was done by fitting an empty two-level random intercept model with adolescents at level-1 and neighbourhoods at level-2, with no covariates (Robson and Pevalin 2015). These models assume a two-level structure where adolescents are *only* nested in neighbourhoods (ignoring schools). Second, a two-level model was run with schools at level-2 (ignoring neighbourhoods) (Dunn et al. 2015). Third, in a cross-classified model, individual adolescents were grouped simultaneously into two non-nested contexts (neighbourhood and school). A variance partition coefficient (VPC) was calculated to estimate the proportion of variance in drinking outcomes that are attributed to neighbourhoods and schools.

A second series of models was conducted to address Research Question 2. Only individuals with complete data on all covariates were included in multivariable models. Model 1 represents a two-level neighbourhood model which included individual socio-demographic factors. Model 2 also included alcohol outlet density and urban/rurality. Model 3 added neighbourhood deprivation. Models 4 and 5 added neighbourhood-level social cohesion and neighbourhood disorder, respectively. Model 6 included neighbourhood social cohesion and neighbourhood disorder together. Model 7 added individual perceptions. Model 8 included a cross-classified specification for school-level variation to ensure associations noted were indeed at the neighbourhood level. The sample was reduced to drinkers when examining weekly drinking and drunkenness.

Variance inflation factor values were below 3 for all independent variables indicating that multicollinearity was not a concern (O’brien 2007). All models were conducted using runmlwin (Leckie and Charlton 2013) via Stata v13 and MLwiN v3.01 with Bayesian estimation procedures as implemented by Markov chain Monte Carlo (MCMC) methods. Because no previous knowledge was assumed, the MLwiN default diffuse prior distributions were used for all estimates. Initial values were derived from an iterative generalised least squares algorithm, and Metropolis–Hastings sampling was used (Browne 2017; Leckie and Charlton 2013). Odds ratios are reported with 95% credible intervals and *p* values. Bayesian deviance information criterion (DIC) was used to test for improvement of model fit, with lower values indicating better fit; generally, a difference of 5 is considered a substantial improvement (Khana et al. 2018; Spiegelhalter et al. 2002). This method was best suited to these analyses as it is appropriate for low numbers of respondents in higher levels of a multilevel model and because maximum likelihood methods are found to be inefficient for cross-classified models (De Clercq et al. 2014; Leckie and Charlton 2013).

## Results

### Participant characteristics

Table 1 outlines the characteristics of the sample. The majority of adolescents had drunk alcohol (83%), while almost half of the respondents (45%) had been drunk twice or more. Twenty-seven per cent of the respondents were weekly drinkers.

**Table 1:** Descriptive statistics of the study sample from the Scottish Health Behaviour in School-aged Children survey, 2010 (*n* = 1558)

Characteristics	Valid *n*	Mean (SD)/*n* (%)	Minimum	Maximum
*Demographics and family characteristics*				
Age	1554	15.55 (0.33)	13.25	16.67
Male	1558	772 (50%)		
White	1558	1515 (97%)		
*Family affluence*	1558			
Low		496 (32%)		
Medium		479 (31%)		
High		583 (37%)		
*Family structure*	1530			
Both parents		1080 (71%)		
Single parent		274 (18%)		
Stepfamily/other		176 (11%)		
*Individual neighbourhood perceptions*				
Perceived neighbourhood disorder^a^	1516	4.92 (1.53)	3	9
Perceived social cohesion^a^	1522	11.98 (2.59)	3	15
*Residential characteristics*				
*Neighbourhood deprivation*	1558			
1 (most deprived)		343 (22%)		
2		358 (23%)		
3		461 (30%)		
4 (least deprived)		396 (25%)		
*Urban/rurality*	1554			
Large urban		263 (17%)		
Other urban		267 (17%)		
Accessible small town		193 (12%)		
Accessible rural		241 (15%)		
Remote small town		198 (13%)		
Remote rural		392 (25%)		
Off-trade alcohol outlets (800 m)	1557	1.59 (1.87)	0	14.25
On-trade alcohol outlets (800 m)	1557	2.91 (4.17)	0	38.31
Neighbourhood-level disorder^b^	1488	− 0.01 (0.14)	− 0.27	0.37
Neighbourhood-level social cohesion^c^	1506	0.04 (0.25)	− 0.61	0.64
*Alcohol use*				
Have ever drunk	1550	1281 (83%)		
Drink weekly	1553	414 (27%)		
Drunk twice or more	1545	689 (45%)		

*SD* Standard deviation

^a^If less than half of the items were missing, mean person imputation was used. This occurred in < 1% of cases

^b^At the neighbourhood level, mean = 0 for 191 neighbourhoods

^c^At the neighbourhood level, mean = 0 for 194 neighbourhoods

### Empty models

For ever drank, weekly drinking, and drunkenness, neighbourhood accounts for 9.7%, 5.7%, and 3.6% of the variation, respectively, when ignoring school-level variation. This was reduced to 7.6%, 5.0%, and 1.0%, respectively, when accounting for school-level variation. For having ever drank and weekly drinking, the DIC was lowest in the cross-classified model compared to the two-level models, suggesting best fit when including both levels. For drunkenness, the DIC was only slightly lower in the cross-classified model, compared to the school-only model (see Supplementary Material).

### Multivariable models

Full results of Models 1–8 for the three drinking outcomes can be found in the Supplementary Material. Models 6–7 are presented in Tables 2 and 3.

**Table 2:** Having ever drunk regressed on neighbourhood and individual measures from the Scottish Health Behaviour in School-aged Children survey, 2010 (95% credible intervals) (*n* = 1457; intermediate data zones *n* = 190; schools *n* = 152)

Variable	Model 6		Model 7	
	OR (95% credible intervals)	*p* value	OR (95% credible intervals)	*p* value
Sex (male)	1.01 (0.74, 1.34)	0.971	1.02 (0.75, 1.35)	0.959
Age	2.04 (1.30, 2.92)	**0.001**	1.83 (1.37, 2.49)	< **0.001**
Family structure (reference: both parents)				
Single parent	1.37 (0.89, 2.04)	0.173	1.31 (0.84,1.96)	0.256
Stepfamily/other	2.05 (1.16, 3.49)	**0.016**	2.00 (1.13, 3.40)	**0.021**
Family affluence (reference: low)				
Medium	1.50 (1.00, 2.15)	**0.048**	1.50 (1.00, 2.18)	0.051
High	1.51 (1.02, 2.15)	**0.036**	1.51 (1.02, 2.16)	**0.041**
Ethnicity (white)	3.06 (1.31, 5.95)	**0.007**	2.74 (1.17, 5.43)	**0.018**
On-trade licence density	0.97 (0.92, 1.03)	0.307	0.97(0.93, 1.03)	0.322
Off-trade licence density	1.02 (0.90, 1.16)	0.742	1.01 (0.88, 1.15)	0.873
Urban/rurality (reference: large cities)				
Other urban	1.47 (0.85, 2.40)	0.181	1.48 (0.84, 2.44)	0.188
Accessible small towns	2.02 (1.03, 3.58)	**0.038**	2.02 (1.04, 3.62)	**0.042**
Accessible rural	2.46 (1.29, 4.28)	**0.005**	2.50 (1.31, 4.40)	**0.005**
Remote small towns	3.70 (1.80, 6.94)	< **0.001**	3.83 (1.83, 7.21)	< **0.001**
Remote rural	3.64 (1.91, 6.37)	< **0.001**	3.61 (1.87, 6.43)	< **0.001**
Neighbourhood deprivation (reference: 1 most deprived)				
2	1.21 (0.70, 1.95)	0.557	1.26 (0.73, 2.04)	0.450
3	0.94 (0.54, 1.52)	0.728	1.01 (0.58, 1.63)	0.910
4 (least deprived)	1.01 (0.57, 1.66)	0.907	1.05 (0.59, 1.74)	0.964
Neighbourhood social cohesion	0.33 (0.12, 0.75)	**0.011**	0.33 (0.10, 0.80)	**0.017**
Neighbourhood disorder	1.25 (0.22, 4.10)	0.941	0.45 (0.06, 1.62)	0.169
Perceived social cohesion	–		0.99 (0.93, 1.06)	0.806
Perceived disorder	–		1.24 (1.10, 1.40)	**0.001**
Neighbourhood variance	0.30 (0.03, 0.66)		0.31 (0.04, 0.68)	
DIC	1302.86		1291.59	

Burn-in 5000, chain 200,000, *OR* odds ratio, *DIC* deviance information criterion, respondents missing on any predictor or outcome were not included in the models, bold = *p* < 0.05

**Table 3:** Weekly drinking and drunkenness regressed on neighbourhood and individual measures from the Scottish Health Behaviour in School-aged Children survey, 2010, among those who have drank (95% credible intervals) (intermediate data zones *n* = 190; schools *n* = 152)

Variable	Weekly drinking (*n* = 1205)				Drunkenness (*n* = 1198)			
	Model 6		Model 7		Model 6		Model 7	
	OR (95% credible intervals)	*p* value	OR (95% credible intervals)	*p* value	OR (95% credible intervals)	*p* value	OR (95% credible intervals)	*p* value
Sex (male)	1.43 (1.10, 1.84)	**0.007**	1.44 (1.10, 1.86)	**0.008**	0.98 (0.77, 1.24)	0.835	0.99 (0.77, 1.25)	0.879
Age	1.38 (0.80, 2.39)	0.339	1.10 (0.75, 1.66)	0.704	1.25 (0.87, 1.75)	0.266	1.18 (0.79, 1.58)	**0.346**
Family structure (reference: both parents)								
Single parent	1.52 (1.06, 2.10)	**0.021**	1.47 (1.02, 2.04)	**0.038**	1.38 (0.98, 1.89)	0.070	1.36 (0.96, 1.87)	0.082
Stepfamily/other	1.16 (0.76, 1.68)	0.537	1.13 (0.74, 1.65)	0.619	2.04 (1.36, 2.97)	**0.001**	2.02 (1.36, 2.97)	**0.001**
Family affluence (reference: low)								
Medium	1.21 (0.85, 1.67)	0.305	1.25 (0.87, 1.73)	0.236	0.93 (0.67, 1.26)	0.606	0.94 (0.68, 1.26)	0.625
High	1.23 (0.88, 1.68)	0.241	1.26 (0.89, 1.72)	0.209	1.28 (0.93, 1.72)	0.132	1.28 (0.93, 1.71)	0.135
Ethnicity (white)	0.71 (0.28, 1.50)	0.713	0.66 (0.25, 1.42)	0.246	0.68 (0.26, 1.47)	0.282	0.66 (0.25, 1.41)	0.241
On-trade licence density	1.00 (0.96, 1.05)	0.938	1.00 (0.96, 1.05)	0.866	1.03 (0.99, 1.08)	0.180	1.03 (0.99, 1.08)	0.174
Off-trade licence density	1.01 (0.90, 1.13)	0.934	0.99 (0.88, 1.11)	0.876	1.00 (0.90, 1.11)	0.947	0.99 (0.89, 1.10)	0.850
Urban/rurality (reference: large cities)								
Other urban	1.23 (0.73, 1.96)	0.490	1.25 (0.72, 2.01)	0.476	1.09 (0.69, 1.66)	0.771	1.09 (0.69, 1.66)	0.779
Accessible small towns	2.04 (1.13, 3.42)	**0.017**	2.08 (1.14, 3.54)	**0.016**	2.24 (1.31, 3.61)	**0.003**	2.24 (1.31, 3.58)	**0.003**
Accessible rural	1.23 (0.68, 2.06)	0.557	1.28 (0.70, 2.19)	0.474	1.34 (0.81, 2.11)	0.280	1.35 (0.81, 2.13)	0.277
Remote small towns	1.26 (0.69, 2.13)	0.501	1.32 (0.72, 2.25)	0.418	1.41 (0.83, 2.24)	0.221	1.42 (0.84, 2.26)	0.208
Remote rural	1.41 (0.78, 2.35)	0.277	1.39 (0.77, 2.34)	0.302	2.04 (1.23, 3.19)	**0.005**	2.01 (1.21, 3.15)	**0.006**
Neighbourhood deprivation (reference: 1 most deprived)								
2	0.89 (0.59,1.31)	0.518	0.91 (0.60, 1.35)	0.593	0.73 (0.49, 1.06)	0.096	0.75 (0.49, 1.07)	0.115
3	0.76 (0.49, 1.14)	0.173	0.80 (0.51, 1.21)	0.271	0.73 (0.49, 1.06)	0.099	0.74 (0.48, 1.09)	0.126
4 (least deprived)	0.63 (0.39, 0.97)	**0.034**	0.64 (0.40, 1.00)	**0.048**	0.70 (0.48, 1.04)	0.072	0.70 (0.45, 1.04)	0.080
Neighbourhood social cohesion	1.07 (0.48, 2.10)	0.989	1.38 (0.56, 2.88)	0.579	0.88 (0.42, 1.62)	0.580	0.88 (0.39, 1.69)	0.590
Neighbourhood disorder	2.31 (0.58, 6.44)	0.291	1.23 (0.25, 3.74)	0.965	3.50 (1.00, 9.10)	0.051	2.36 (0.60, 6.54)	0.272
Perceived social cohesion	–		0.95 (0.89, 1.01)	0.082			1.00 (0.94, 1.06)	0.958
Perceived disorder	–		1.14 (1.03, 1.27)	**0.011**			1.09 (0.99, 1.19)	0.090
Neighbourhood variance	0.12 (0.00, 0.40)		0.17 (0.00, 0.46)		0.06 (0.00, 0.22)		0.05 (0.00, 0.22)	
DIC	1513.15		1499.86		1631.51		1631.68	

Burn-in 5000, Chain 200,000, *OR* odds ratio, *DIC* deviance information criterion, respondents missing on any predictor or outcome were not included in the models, bold = *p* < 0.05

Urban/rurality showed a clear gradient in alcohol use (Table 2—Model 7); those in remote and rural regions had higher odds of having ever drunk than those in large cities, while those in smaller urban areas were not significantly different in terms of ever drinking (*p* > 0.05). A significant association was present for neighbourhood social cohesion on having ever drunk alcohol (odds ratio = 0.33, *p* = 0.017), in fully adjusted models. Including this measure also improved model fit compared to the null model (DIC = 1301.69 vs. 1304.15) (see Supplementary Material). In fully adjusted models (Model 7), no significant associations were found for AODs or neighbourhood disorder with having ever drank (*p* > 0.05); however, individual perceived disorder was associated with having ever drank, (odds ratio = 1.24, *p* = 0.001).

Among those who had ever drank, those residing in the least deprived areas had reduced odds of weekly drinking compared to those in the most deprived areas (odds ratio = 0.64, *p* = 0.048), in fully adjusted models (Table 3—Model 7). Additionally, those in accessible small towns had higher odds of weekly drinking than those in large urban areas (odds ratio = 2.08, *p* = 0.016). No significant associations were found for AODs, neighbourhood disorder, or neighbourhood social cohesion (*p* > 0.05). Individual perceived disorder was associated with weekly drinking (odds ratio = 1.14, *p* = 0.011).

Turning now to drunkenness, among those who had ever drank, those in accessible small towns (odds ratio = 2.24, *p* = 0.003) and remote rural areas (odds ratio = 2.01, *p* = 0.006) had higher odds of drunkenness than those in large urban areas (Table 3—Model 7), in the fully adjusted models. Those residing in areas of lower deprivation had significantly reduced odds of drunkenness; however, this relationship became non-significant when accounting for neighbourhood disorder (see Supplementary Material). Neighbourhood-level disorder was associated with increased odds of drunkenness (see Supplementary Material); however, this relationship was no longer significant when accounting for neighbourhood social cohesion and individual neighbourhood perceptions (Models 6–7).

For all outcomes, the associations in Model 7 were still observed after accounting for school-level variation (see Supplementary Material). Sensitivity analysis using different distance bands to measure AODs did not influence main findings from the models.

Because the data are spatially distributed, a global Moran’s I was calculated on the IDZ residuals from Model 8 to detect whether spatial autocorrelation was present, which would violate the assumption of independence of error-terms. The Moran’s I statistic was not significant (*p* > 0.05) (see Supplementary Material), indicating no spatial clustering in the model residuals (Anselin and Griffith 1988).

## Discussion

This study used multilevel analysis to examine associations of neighbourhood characteristics with adolescent drinking behaviours. The results are strengthened by the inclusion of the school-level; thus, testing whether the findings are, in fact, overestimated due to the omission of the school-level variance. Results show that having ever consumed alcohol and weekly alcohol use varied by neighbourhood and are in line with a study of US adolescents that found significant variance of alcohol misuse at the neighbourhood level but not the school level (Ennett et al. 2008). However, school explained a greater amount of variance in drunkenness. This may be due to binge drinking being more influenced by shared peer culture experienced at school (Kuntsche and Jordan 2006).

The more remote and rural the area an adolescent resided in, the higher the odds of having ever drank. Other studies in Scotland have found an urban/rural difference (using a dichotomous measure) in whether adolescents had ever drunk alcohol (The Scottish Government 2016). Our current research found that among drinkers, those living in accessible small towns had higher odds of weekly drinking and drunkenness and those in remote rural areas had higher odds of drunkenness. This supports the principle that more detailed classifications of urban/rural are necessary, as suggested by Dixon and Chartier (2016). Additionally, the results reflect previous research on adolescent illicit substance and tobacco use, which maintain that adolescent substance use in Scotland is not concentrated in urban areas (Forsyth and Barnard 1999; Levin et al. 2014). The associations related to urban/rurality remained unexplained after controlling for neighbourhood social conditions and AOD, indicating that there may be other reasons for these inequalities. It may be that in rural areas and accessible small towns, adolescent alcohol use may be normalised and used as a form of “cultural capital” (Kloep et al. 2001).

It is important to note that the sample was made up of 15-year-olds; therefore, findings of an urban/rural gradient in having ever drunk represent a more delayed initiation to drinking but do not necessarily translate to lifetime abstention throughout adulthood. Conversely, many studies have found that, among adults, those in urban areas have higher rates of alcohol use compared to those in rural areas (Dixon and Chartier 2016; Slutske et al. 2016). Comprehension of different drinking trajectories across the life course, in terms of urban/rurality, is needed to explain this pattern.

Those living in an area of low deprivation had lower odds of weekly drinking, but not having ever drunk, or drunkenness (in fully adjusted models). Based on these findings, a potential explanation for the mixed results found in previous studies of neighbourhood socio-economics and adolescent alcohol use could be due to differing alcohol outcomes. Our results are in accordance with other research that found a relationship with neighbourhood deprivation and regular drinking among adolescents in Scotland (Petrou and Kupek 2018). The current study strengthens that evidence in that it adjusts for other neighbourhood conditions and family factors and confirms that this relationship holds.

Neighbourhood social cohesion was negatively associated with having ever drunk by S4; however, among drinkers, there was no association with alcohol use drinking behaviours. This is counter to findings from an urban US study that found neighbourhood collective efficacy did not influence adolescent alcohol use (Fagan et al. 2015). This may be due to their measures of the social environment originating from adults rather than adolescents. Conversely, Jackson et al. (2016) found collective efficacy, as measured by adolescents, was associated with adolescent drinking outcomes in an urban sample. Our findings support theories which argue that positive social connections discourage adolescent alcohol use; however, the association is limited to alcohol initiation. More research is needed to determine if creating more cohesive communities could reduce the likelihood of adolescents commencing alcohol use.

Unlike previous studies of Scottish adult populations (Shortt et al. 2018), we did not find an association between AOD and adolescent drinking outcomes. This may be because 15-year-olds are unlikely to purchase alcohol directly from retailers due to Scotland’s age restrictions and regulations (The Scottish Government 2016). It is noteworthy that the measure of on-trade outlets did not distinguish between establishment types. These may have differing impacts for adolescents as, unlike adults, they are restricted in terms of alcohol access in these venues. Some establishments would primarily be drinking establishments and may influence social norms in the neighbourhood, while other establishments may serve as a source of entertainment with alcohol consumption not being the primary activity. Moreover, the impact of AOD may only be observed over time after repeat exposure; longitudinal studies are needed to examine this possibility.

This study has several strengths, including having a boosted sample of non-urban youth, accounting for a variety of theoretically important neighbourhood conditions, and adjusting for school-level variation. Some limitations are worth consideration. First, this study is cross-sectional, so causation cannot be inferred. Additionally, IDZs were used to represent neighbourhoods. However, this is an administrative unit and may not correspond to the respondents’ understandings of their neighbourhood boundaries. Moreover, the neighbourhood-level social cohesion and disorder measures are derived from the same adolescents who reported their drinking behaviours; therefore, this study is at risk of same-source bias (Jackson et al. 2016). Further, we were unable to examine family structures that did not include a biological parent due to small numbers of students reporting these family compositions. Future studies designed to explicitly examine alcohol consumption among young people in alternative family situations are required. Finally, the focus of this study was on neighbourhood characteristics. Future studies may examine school characteristics. This is of particular interest for drunkenness given the greater proportion of variance accounted for by school compared to neighbourhood.

Despite these limitations, the results have important implications for public health strategies. Efforts that are targeted to rural areas, small towns, and neighbourhoods with low social cohesion are needed, given higher rates of adolescent alcohol use. Additionally, services and interventions should be directed at regions of high deprivation in Scotland, due to the higher rates of regular alcohol use. Future work is needed to develop and evaluate intervention and prevention approaches targeted to neighbourhoods at greatest risk.

## Electronic supplementary material

Below is the link to the electronic supplementary material.

Supplementary material 1 (PDF 773 kb)
