# Supplementary material for: The neighbourhood social environment and alcohol use among urban and rural Scottish adolescents
Source: Int J Public Health. 2018 Dec 3;64(1):95–105. doi: 10.1007/s00038-018-1181-8 (PMC6353998; doi:10.1007/s00038-018-1181-8)
Supplement: Supplementary file 1 — Supplementary material 1 (PDF 773 kb) [file 38_2018_1181_MOESM1_ESM.pdf]

Journal: International Journal of Public Health

Title: The neighbourhood social environment and alcohol use among urban and rural Scottish adolescents

Kernel density estimation (KDE)

Kernel density estimation (KDE) for spatial point location data produces a smooth, continuous geographic surface for which every location in the study area is assigned a value which can then be used as an independent or dependent variable in statistical models (Carlos et al. 2010). The density of points is calculated using a pre-specified cell size, a kernel function, and a bandwith (a circle with a radius centred on the focal location) such that the surface is highest above the case (outlet location) and zero at the specified bandwidth from the outlet. The centre of this cell size receives the density value (Chang, 2006). It is important to note that increasing the bandwidth produces a greater smoothing effect (the surface becomes more generalised by reducing the number and magnitude of local maxima) from the original data distribution. The selection of a bandwidth distance that is too large or too small, may over or under-smooth the data (Carlos et al. 2010, Chainey 2013). Therefore, sensitivity analysis is important. Alcohol outlet density has been previously examined using KDE (for example see Carlos et al. 2010).

References:

Carlos, H. A., et al. (2010) "Density estimation and adaptive bandwidths: a primer for public health practitioners." Int J Health Geogr 9: 39

Chainey, S. P. (2013) "Examining the influence of cell size and bandwidth size on kernel density estimation crime hotspot maps for predicting spatial patterns of crime." Bull Geographical Soc Liege 60: 7-19

Chang, K. (2006) *Geographic information systems*. John Wiley & Sons, Ltd.

Supplementary Table 1: Empty models examining drinking behaviours across neighbourhoods and schools, Scottish Health Behaviour in School-aged Children Survey, 2010 (95% credible intervals)

	Neighbourhood only	School only	Cross-classified
Have ever drank (n=1550)			
Neighbourhood level variance	0.353 (0.083,0.677)*		0.281 (0.011,0.631)
School level variance		0.303 (0.072,0.608)*	0.147 (0.002,0.475)
Neighbourhood % of variance accounted for	9.7		7.6
School % of variance accounted for		8.4	3.9
DIC	1411.31	1414.74	1409.31
Weekly drinking (n=1553)			
Neighbourhood level variance	0.199 (.007,0.445)		0.177 (0.005,0.423)
School level variance		0.114 (0.001,0.318)	0.059(0.001,0.248)
Neighbourhood % of variance accounted for	5.7		5.0
School % of variance accounted for		3.3	1.7
DIC	1791.45	1797.58	1790.50
Lifetime drunkenness (n=1545)			
Neighbourhood level variance	0.123 (0.002,0.310)		0.034 (0.001,0.173)
School level variance		0.155 (0.002,0.346)	0.146 (0.011,0.329)
Neighbourhood % of variance accounted for	3.6		1.0
School % of variance accounted for		4.7	4.2
DIC	2119.55	2110.63	2110.48

DIC= Deviance Information Criterion; Burn-in 5,000; chain 200,000; *p<0.05; Significance determined by z-score probability in multilevel models; DIC is used to examine for model fit improvement in cross-classified models by comparing to non-cross-classified models.

Supplementary Table 2: Having ever drank regressed on neighbourhood and individual measures (95% credible intervals) (n=1,457; Intermediate Data Zones n=190; Schools n=152), Scottish Health Behaviour in School-aged Children Survey, 2010

Variable	Model 1	Model 2	Model 3	Model 4	Model 5	Model 6	Model 7	Model 8
Sex (male)	1.00 (0.74.1.29)	1.01 (0.74.1.33)	1.01 (0.74.1.34)	1.00 (0.74.1.33)	1.01 (0.74.1.33)	1.01 (0.74.1.34)	1.02 (0.75,1.35)	1.02 (0.75,1.36)
Age	1.98 (1.26,2.80)***	1.87 (1.13,3.12)**	1.76 (1.07,2.87)**	2.12 (1.19,2.96)**	2.00(1.37,2.78)***	2.04 (1.30,2.92)**	1.83(1.37,2.49)***	1.89 (1.25,2.78)**
Family Structure (Ref: both parents)								
single parent	1.45 (0.95,2.14)	1.42 (0.96,1.98)	1.38 (0.90,2.07)	1.37 (0.89,2.05)	1.40 (0.90,2.08)	1.37 (0.89,2.04)	1.31 (0.84,1.96)	1.30 (0.84,1.96)
step-family/other	2.20 (1.24,3.72)**	2.09 (1.18,3.55)*	2.01 (1.14,3.43)*	2.05 (1.15,3.51)*	2.04 (1.15,3.48)*	2.05 (1.16,3.49)*	2.00 (1.13,3.40) *	2.00 (1.13,3.42)*
Family Affluence (Ref: low)								
medium	1.46 (0.98,2.08)	1.42 (0.96,2.04)	1.46 (0.99,2.11)	1.49 (1.00,2.15)	1.48 (0.99,2.14)	1.50 (1.00,2.15)*	1.50 (1.00,2.18)	1.52 (1.01,2.21)*
high	1.39 (0.96,1.94)	1.41 (0.97,1.98)	1.48 (1.01,2.08)*	1.51 (1.03,2.15)*	1.49 (1.01,2.12)*	1.51 (1.02,2.15)*	1.51 (1.02,2.16)*	1.53 (1.03,2.19)*
Ethnicity (white)	3.39 (1.51, 6.48)**	2.96 (1.30,5.79)**	2.94 (1.31,5.71)*	3.00 (1.32,5.82)**	3.09 (1.37,5.90)**	3.06 (1.31,5.95)**	2.74(1.17,5.43) *	2.78 (1.18,5.52)*
On trade licence density		0.97 (0.93,1.02)	0.98 (0.93,1.03)	0.97 (0.93,1.03)	0.97 (0.93,1.03)	0.97 (0.92,1.03)	0.97(0.93,1.03)	0.97 (0.92,1.03)
Off trade license density		1.06 (0.93,1.20)	1.04 (0.91,1.17)	1.02 (0.90,1.16)	1.04 (0.91,1.18)	1.02 (0.90,1.16)	1.01 (0.88,1.15)	1.02 (0.89,1.16)
Urban/rurality (Ref: large cities)								
other urban		1.42 (0.83,2.30)	1.39 (0.80,2.29)	1.46 (0.84,2.40)	1.40 (0.81,2.30)	1.47 (0.85,2.40)	1.48 (0.84,2.44)	1.45 (0.81,2.42)
accessible small towns		1.83 (0.97,3.19)	1.79 (0.94,3.13)	1.99 (1.04,3.52)*	1.71 (0.89,3.05)	2.02 (1.03,3.58)*	2.02 (1.04,3.62)*	2.12 (1.04,3.91)*

Electronic supplementary material (ESM)

Variable	Model 1	Model 2	Model 3	Model 4	Model 5	Model 6	Model 7	Model 8
accessible rural		1.95 (1.06,3.29)*	2.02 (1.08,3.47)*	2.43 (1.27,4.19)**	2.06 (1.09,3.57)*	2.46 (1.29,4.28) **	2.50 (1.31,4.40) **	2.53 (1.31,4.51)**
remote small towns		3.23 (1.61,5.97)**	3.07 (1.50,5.71)**	3.66(1.79,6.84)***	3.12 (1.53,5.82)**	3.70(1.80,6.94)***	3.83(1.83,7.21)***	4.07(1.91,7.88)***
remote rural		2.59 (1.46,4.29)**	2.60 (1.43,4.39)**	3.59(1.88,6.33)***	2.71 (1.48,4.61)**	3.64(1.91,6.37) ***	3.61(1.87,6.43)***	3.60(1.83,6.50)***
Neighbourhood deprivation (Ref: 1 most deprived)								
2			1.03 (0.61,1.63)	1.20 (0.70,1.92)	1.09 (0.65,1.75)	1.21 (0.70,1.95)	1.26 (0.73, 2.04)	1.30 (0.75,2.13)
3			0.78 (0.46,1.23)	0.94 (0.55,1.49)	0.85 (0.50,1.37)	0.94 (0.54,1.52)	1.01 (0.58, 1.63)	1.02 (0.59,1.66)
4 least deprived			0.81 (0.47,1.28)	0.99 (0.57,1.61)	0.88 (0.50,1.44)	1.01 (0.57,1.66)	1.05 (0.59,1.74)	1.09 (0.60,1.81)
Neighbourhood social cohesion				0.34 (0.13,0.71)**		0.33 (0.12,0.75)*	0.33 (0.10,0.80)*	0.33 (0.10,0.77)*
Neighbourhood disorder					2.57 (0.53,7.92)	1.25 (0.22,4.10)	0.45 (0.06, 1.62)	0.45 (0.06,1.61)
Perceived social cohesion							.99 (0.93,1.06)	0.99 (0.92,1.06)
Perceived disorder							1.24(1.10,1.40)***	1.23 (1.09,1.39)**
Neighbourhood variance	0.36 (0.07,0.72)	0.30 (0.03,0.66)	0.32 (0.01,0.69)	0.29 (0.02,0.65)	0.32 (0.03,0.69)	0.30 (0.03,0.66)	0.31 (0.04,0.68)	0.19 (0.00,0.60)
School variance								0.16 (0.00,0.52)
DIC	1304.15	1302.91	1307.21	1301.69	1306.65	1302.86	1291.59	1292.85
Residual Moran's I								0.016 (p=0.505)

Burn-in 5,000; chain 200,000; DIC=Deviance Information Criterion; Respondents missing on any predictor or outcome were not included in the models; *=p<0.05, **=p<0.01, ***p<0.001

Supplementary Table 3: Weekly alcohol consumption regressed neighbourhood and individual measures, among lifetime drinkers (95% credible intervals) (n=1,205; Intermediate Data Zones n=190; Schools 148), Scottish Health Behaviour in School-aged Children Survey, 2010

Predictor variable	Model 1	Model 2	Model 3	Model 4	Model 5	Model 6	Model 7	Model 8
Sex (male)	1.42 (1.10,1.83)**	1.45 (1.11,1.86)**	1.43 (1.10,1.83)**	1.43 (1.10,1.83)**	1.43 (1.10,1.84)**	1.43 (1.10,1.84)**	1.44 (1.10,1.86)**	1.44 (1.09,1.86)**
Age	1.23 (0.78,1.64)	1.19 (0.67,1.98)	1.13 (0.71,1.76)	1.23 (0.92,1.63)	1.26 (0.86,1.70)	1.38 (0.80,2.39)	1.10 (0.75,1.66)	1.22 (0.82,1.54)
Family Structure (Ref: both parents)								
single parent	1.62 (1.14,2.23)**	1.52 (1.11,2.18)*	1.50 (1.05,2.07)*	1.51 (1.06,2.09)*	1.51 (1.06,2.09)*	1.52 (1.06,2.10)*	1.47 (1.02,2.04)*	1.47 (1.03,2.04)*
step family/other	1.23 (0.81,1.78)	1.20 (0.79,1.74)	1.14 (0.75,1.66)	1.15 (0.76,1.68)	1.15 (0.76,1.67)	1.16 (0.76,1.68)	1.13 (0.74,1.65)	1.13 (0.74,1.66)
Family Affluence (Ref: low)								
medium	1.12 (0.80,1.53)	1.14 (0.81,1.57)	1.19 (0.84,1.63)	1.19 (0.84,1.64)	1.20 (0.85,1.65)	1.21 (0.85,1.67)	1.25 (0.87,1.73)	1.25 (0.88,1.73)
high	1.10 (0.79,1.49)	1.14 (0.82,1.55)	1.22 (0.87,1.66)	1.22 (0.87,1.67)	1.22 (0.87,1.67)	1.23 (0.88,1.68)	1.26 (0.89,1.72)	1.26 (0.89,1.73)
Ethnicity (white)	0.72 (0.27, 1.59)	0.69 (0.27, 1.42)	0.66 (0.24,1.40)	0.68 (0.26,1.46)	0.72 (0.28, 1.61)	0.71 (0.28, 1.50)	0.66 (0.25, 1.42)	0.66 (0.25, 1.46)
On trade licence density		1.00 (0.96,1.05)	1.01 (0.96,1.05)	1.01 (0.96,1.05)	1.00 (0.96,1.05)	1.00 (0.96,1.05)	1.00 (0.96,1.05)	1.01 (0.96,1.05)
Off trade license density		1.04 (0.93,1.16)	1.00 (0.90,1.12)	1.00 (0.89,1.12)	1.01 (0.90,1.12)	1.01 (0.90,1.13)	0.99 (0.88,1.11)	0.99 (0.88,1.11)
Urban/rurality (Ref: Large cities)								

Electronic supplementary material (ESM)

Predictor variable	Model 1	Model 2	Model 3	Model 4	Model 5	Model 6	Model 7	Model 8
other urban		1.24 (0.73,1.99)	1.21 (0.72,1.92)	1.22 (0.72,1.94)	1.23 (0.73,1.95)	1.23 (0.73,1.96)	1.25 (0.72,2.01)	1.24 (0.72,2.01)
accessible small towns		2.19 (1.24,3.66) **	2.11 (1.22,3.47) **	2.16 (1.23,3.57) **	2.03 (1.16,3.35)*	2.04 (1.13,3.42)*	2.08 (1.14,3.54)*	2.05 (1.12,3.49)*
accessible rural		1.15 (0.65,1.92)	1.19 (0.68,1.97)	1.23 (0.69,2.06)	1.22 (0.69,2.02)	1.23 (0.68,2.06)	1.28 (0.70,2.19)	1.26 (0.69,2.15)
remote small towns		1.34 (0.76,2.24)	1.23 (0.71,2.03)	1.27 (0.71,2.11)	1.26 (0.71,2.09)	1.26 (0.69,2.13)	1.32 (0.72, 2.25)	1.30 (0.70, 2.24)
remote rural		1.34 (0.80,2.15)	1.32 (0.79,2.10)	1.40 (0.79,2.30)	1.40 (0.82,2.26)	1.41 (0.78,2.35)	1.39 (0.77,2.34)	1.40 (0.78,2.36)
Neighbourhood deprivation (Ref: 1 most deprived)								
2			0.87 (0.57,1.26)	0.89 (0.59,1.29)	0.90 (0.60,1.32)	0.89 (0.59,1.31)	0.91 (0.60,1.35)	0.92 (0.60,1.35)
3			0.72 (0.47,1.06)	0.74 (0.48,1.09)	0.77 (0.50,1.14)	0.76 (0.49,1.14)	0.80 (0.51,1.21)	0.81 (0.51,1.22)
4 least deprived			0.59 (0.37,0.89)*	0.60 (0.38,0.91)*	0.64 (0.40,0.97)*	0.63 (0.39,0.97)*	0.64 (0.40,1.00)*	0.64 (0.39,1.00)*
Neighbourhood social cohesion				0.90 (0.44,1.67)		1.07 (0.48,2.10)	1.38 (0.56,2.88)	1.35 (0.55,2.84)
Neighbourhood disorder					2.27 (0.65,5.90)	2.31 (0.58, 6.44)	1.23 (0.25, 3.74)	1.20 (0.25, 3.61)
Perceived social cohesion							.95 (0.89,1.01)	.95 (0.90,1.01)
Perceived disorder							1.14 (1.03,1.27)*	1.15 (1.03,1.27)*
Neighbourhood variance	0.18 (0.01,0.44)	0.17 (0.00,0.44)	0.11 (0.00,0.37)	0.12 (0.00,0.38)	0.12 (0.00,0.38)	0.12 (0.00,0.40)	0.17 (0.00,0.46)	0.14 (0.00,0.42)
School variance								0.05 (0.00,0.22)

Electronic supplementary material (ESM)

Predictor variable	Model 1	Model 2	Model 3	Model 4	Model 5	Model 6	Model 7	Model 8
DIC	1503.53	1509.55	1509.10	1508.99	1508.47	1513.15	1499.86	1500.13
Residual Moran's I								-0.031 (p=0.406)

Burn-in 5,000; chain 200,000; DIC=Deviance Information Criterion; Respondents missing on any predictor or outcome were not included in the models; *=p<0.05, **=p<0.01, ***p<0.001

Electronic supplementary material (ESM)

Supplementary Table 4: Drunkenness regressed on neighbourhood and individual measures, among current drinkers (95% credible intervals) (n=1,198; Intermediate Data Zones n=190; Schools 148), Scottish Health Behaviour in School-aged Children Survey, 2010

**Predictor variable**	**Model 1**	**Model 2**	**Model 3**	**Model 4**	**Model 5**	**Model 6**	**Model 7**	**Model 8**
Sex (male)	0.98 (0.77,1.23)	0.99 (0.77,1.25)	0.98 (0.77,1.24)	0.98 (0.77,1.23)	0.98 (0.77,1.23)	0.98 (0.77,1.24)	0.99 (0.77,1.25)	1.00 (0.78,1.27)**
Age	1.24 (0.76,2.06)	1.00 (0.57,1.54)	1.22 (0.76,1.92)	1.24 (0.78,1.90)	1.29 (0.79,1.93)	1.25 (0.87,1.75)	1.18 (0.79,1.58)	1.16 (0.81,1.53)
Family Structure (Ref: both parents)								
single parent	1.50 (1.08,2.05)*	1.42 (1.01,1.94)*	1.36 (1.06,1.86)	1.36 (0.97,1.86)	1.37 (0.97,1.89)	1.38 (0.98,1.89)	1.36 (0.96,1.87)	1.35 (1.03,1.87)
step family/other	2.17 (1.46,3.13) ***	2.07 (1.38,2.99)***	2.02 (1.35,2.93)**	2.01 (1.35,2.92)**	2.03 (1.36,2.94)**	2.04 (1.36,2.97)**	2.02 (1.36,2.97)**	2.09 (1.38, 3.08)*
Family Affluence (Ref: low)								
medium	0.87 (0.63,1.16)	0.86 (0.63,1.16)	0.91 (0.66,1.22)	0.91 (0.66,1.23)	0.93 (0.67,1.26)	0.93 (0.67,1.26)	0.94 (0.68,1.26)	0.92 (0.66,1.26)
high	1.10 (0.81,1.46)	1.16 (0.85,1.54)	1.24 (0.91,1.66)	1.26 (0.92,1.68)	1.27 (0.93,1.71)	1.28 (0.93,1.72)	1.28 (0.93,1.71)	1.27 (0.92,1.72)
Ethnicity (white)	0.72 (0.25, 1.61)	0.63 (0.23, 1.33)	0.60 (0.22,1.30)	0.64 (0.24,1.33)	0.65 (0.23,1.40)	0.68 (0.26, 1.47)	0.66 (0.25, 1.41)	0.63 (0.23, 1.35)
On trade licence density		1.03 (0.99,1.08)	1.04 (0.99,1.08)	1.03 (0.99,1.08)	1.03 (0.99,1.07)	1.03 (0.99,1.08)	1.03 (0.99,1.08)	1.03 (0.99,1.08)
Off trade license density		1.04 (0.94,1.15)	0.99 (0.90,1.11)	0.99 (0.89,1.10)	1.00 (0.90,1.11)	1.00 (0.90,1.11)	0.99 (0.89,1.10)	0.99 (0.89,1.11)
Urban/rurality (Ref: Large cities)								
other urban		1.07 (0.68,1.60)	1.05 (0.66,1.58)	1.08 (0.68,1.63)	1.07 (0.68,1.62)	1.09 (0.69,1.66)	1.09 (0.69,1.66)	1.09 (0.69,1.65)
accessible small towns		2.41 (1.44,3.81) **	2.35 (1.40,3.71) **	2.44 (1.46,3.86) **	2.16 (1.28,3.43) **	2.24 (1.31,3.61) **	2.24 (1.31,3.58) **	2.23 (1.24,3.72) **
accessible rural		1.19 (0.73,1.83)	1.25 (0.77,1.92)	1.33 (0.81,2.09)	1.28 (0.78,2.01)	1.34 (0.81,2.11)	1.35 (0.81,2.13)	1.39 (0.80,2.25)

Electronic supplementary material (ESM)

Predictor variable	Model 1	Model 2	Model 3	Model 4	Model 5	Model 6	Model 7	Model 8
remote small towns		1.39 (0.85,2.16)	1.31 (0.79,2.05)	1.40 (0.84,2.21)	1.35 (0.81,2.11)	1.41 (0.83,2.24)	1.42 (0.84,2.26)	1.46 (0.82,2.42)
remote rural		1.67 (1.07,2.49)*	1.74 (1.10,2.62)*	1.99 (1.21,3.09)**	1.91 (1.19,2.91)**	2.04 (1.23,3.19)**	2.01 (1.21,3.15)**	2.10 (1.21,3.43)**
Neighbourhood deprivation (Ref: 1 most deprived)								
2			0.68 (0.45,0.97)*	0.72 (0.48,1.03)	0.72 (0.48,1.03)	0.73 (0.49,1.06)	0.75 (0.49,1.07)	0.74 (0.48,1.08)
3			0.64 (0.42,0.92) *	0.68 (0.45,0.98) *	0.71 (0.47,1.04)	0.73 (0.49,1.06)	0.74 (0.48,1.09)	0.73 (0.47,1.09)
4 least deprived			0.60 (0.39,0.87)**	0.64 (0.42,0.94)*	0.68 (0.44,1.00)	0.70 (0.48,1.04)	0.70 (0.45,1.04)	0.69 (0.43,1.04)
Neighbourhood social cohesion				0.67 (0.35,1.13)		0.88 (0.42,1.62)	0.88 (0.39,1.69)	0.86 (0.37,1.72)
Neighbourhood disorder					3.78 (1.25,9.05)*	3.50 (1.00, 9.10)	2.36 (0.60, 6.54)	2.03 (0.46, 5.80)
Perceived social cohesion							1.00 (0.94,1.06)	1.00 (0.94,1.06)
Perceived disorder							1.09 (0.99,1.19)	1.09 (0.99,1.20)
Neighbourhood variance	0.09 (0.00,0.30)	0.06 (0.00,0.23)	0.04 (0.00,0.19)	0.03 (0.00,0.13)	0.04 (0.00,0.18)	0.06 (0.00,0.22)	0.05 (0.00,0.22)	0.03 (0.00,0.17)
School variance								0.13 (0.00,0.37)
DIC	1646.49	1638.34	1633.95	1633.01	1630.61	1631.51	1631.68	1627.20
Residual Moran's I								0.008 (p=0.675)

Burn-in 5,000; chain 200,000; DIC=Deviance Information Criterion; Respondents missing on any predictor or outcome were not included in the models; *=p<0.05, **=p<0.01, ***p<0.001
